# Peroxiredoxin6 in Endothelial Signaling

**DOI:** 10.3390/antiox8030063

**Published:** 2019-03-13

**Authors:** Priyal Patel, Shampa Chatterjee

**Affiliations:** Institute for Environmental Medicine and Department of Physiology, University of Pennsylvania Perelman School of Medicine, Philadelphia, PA 19104, USA; patpri@pennmedicine.upenn.edu

**Keywords:** phospholipase A2, glutathione peroxidase, reactive oxygen species, redox balance, endothelium, inflammation, diabetes

## Abstract

Peroxiredoxins (Prdx) are a ubiquitous family of highly conserved antioxidant enzymes with a cysteine residue that participate in the reduction of peroxides. This family comprises members Prdx1–6, of which Peroxiredoxin 6 (Prdx6) is unique in that it is multifunctional with the ability to neutralize peroxides (peroxidase activity) and to produce reactive oxygen species (ROS) via its phospholipase (PLA_2_) activity that drives assembly of NADPH oxidase (NOX2). From the crystal structure, a C47 residue is responsible for peroxidase activity while a catalytic triad (S32, H26, and D140) has been identified as the active site for its PLA_2_ activity. This paradox of being an antioxidant as well as an oxidant generator implies that Prdx6 is a regulator of cellular redox equilibrium (graphical abstract). It also indicates that a fine-tuned regulation of Prdx6 expression and activity is crucial to cellular homeostasis. This is specifically important in the endothelium, where ROS production and signaling are critical players in inflammation, injury, and repair, that collectively signal the onset of vascular diseases. Here we review the role of Prdx6 as a regulator of redox signaling, specifically in the endothelium and in mediating various pathologies.

## 1. Introduction

Peroxiredoxins (Prdx) are a family of enzymes that primarily function as antioxidants to scavenge peroxide in biological systems. The six isoforms of mammalian Prdxs (Prdx1 to 6) are distributed across various cellular sites of reactive oxygen species (ROS) production, such as the cytosol, mitochondria, and peroxisomes [1,2]. Prdxs are divided into two subgroups: Those that have one (Prdx6) cysteine (Cys) residue to participate in the redox cycle and those that have two (Prdx1–5). The enzymes Prdx1–5 use thioredoxin as an electron donor in their redox cycle. However, Prdx6 does not use thioredoxin and uses glutathione (GSH) as the reductant instead [3]. Prdxs are small proteins with sizes varying between 22 and 27 KDa; of these, Prdx6 in its native form is a 26 KDa protein and consists of 224 amino acids. The human Prdx6 gene comprises 11,542 base pairs and is located on Chromosome 1. The protein encoded by this gene is a member of the thiol-specific antioxidant protein family. Prdx6 is reported to be expressed in almost all cell types with high expression levels noted in lung endothelial and epithelial cells, lens epithelial cells, hepatocytes, leukocytes, neutrophils, etc. [4,5].

Prdx6 has long been established as a bifunctional enzyme. The antioxidant property, i.e., the peroxidase activity, is dependent on the catalytic Cys at position 47 [6], which is reduced by GSH S-transferase-bound GSH to complete the catalytic cycle [7], while the other enzymatic function of Prdx6 (a calcium-independent phospholipase (PLA_2_) activity) is dependent on a catalytic triad: Ser32, His26, and Asp140 [8], which catalyze the hydrolysis of the acyl group of phospholipids. The PLA_2_ activity of Prdx6 has been reported to play a major role in the metabolism of the phospholipids of lung surfactant [9] and to activate NADPH oxidase [10,11]. Prdx6 is a cytosolic enzyme, but upon stimulation of cells (with inflammatory stimuli), it is phosphorylated and the phosphorylated form translocates to the plasma membrane, where it supports NADPH oxidase activity [5].

Thus Prdx6 is unique in that it possesses both glutathione peroxidase (GPx) and calcium independent phospholipase A_2_ (PLA_2_) activities [9]. Prdx6 is able to act as an antioxidant by limiting oxidative stress by reducing short-chain hydroperoxides through its peroxidase activity. However, the PLA_2_ activity specific to Prdx6 leads to the generation of oxidants [11]. Thus, while the peroxidase activity is crucial in protecting against oxidative stress, the PLA_2_ activity plays an important role in the production of reactive oxygen species (ROS). These two paradoxical abilities of Prdx6 seem to point to a regulatory role of this enzyme in oxidative stress. It is conceivable that Prdx6 acts as a regulator or rheostat to fine tune ROS levels via the two activities, so as to enable optimal ROS levels for maintaining vascular homeostasis.

The lungs are one of the major organs exposed to the environment. The airways are in direct contact with a wide range of chemical and biological components in the atmosphere. Environmental agents also affect the lung vasculature via the alveolar–capillary structure, where pulmonary gas exchange occurs. The lung is also highly vascularized, with the endothelium that lines the lung vessels comprising >30% of the lungs [12]. Moreover, the endothelium, specifically the pulmonary endothelium, is the converging site of inflammation, whereby polymorphonuclear neutrophils (PMN) adhere to the vessel wall, followed by their transmigration into tissue. Reports have established that endothelial redox signaling facilitates lung inflammation via upregulation of adherence and transmigration of PMN, macrophages, and other immune cells [12,13,14].

Endothelial oxidative stress arises as a consequence of an imbalance between the production of ROS and the antioxidant defenses. The increase in ROS (either external or generated by the cell) leads to alteration of cellular proteins and organelles and is a major cause of cell death. Among the antioxidant defenses is Prdx6, which is highly expressed in the lung; indeed both endothelial and non-endothelial cells of the lung express high amounts of Prdx6 [11,15]. In addition to acting as an antioxidant, we reported that Prdx6 also participates in the generation of ROS, specifically in endothelial cells [11]. This review will focus on how these contradictory roles of Prdx6 facilitate the regulation vascular homeostasis and disease.

## 2. Peroxiredoxin 6 in the Endothelium

The endothelium is a critical component of vascular function by virtue of the signaling pathways that are activated in response to both physical forces, associated with blood flow (endothelial mechanotransduction) [16,17] and chemical stimuli (endothelial chemotransduction) [12,18] from chemical toxins, bacterial endotoxins, etc. Prdx6 expression in the endothelium has been reported, and it has been observed showing a steep increase with oxidative pathologies [19]. Examination of the aortic wall of subjects with aortic aneurysm showed a high expression of Prdx6 in the atherosclerotic plaques [20]. Lack of Prdx6 in the endothelium has been noted to increase sensitivity to oxidative stress. Conversely, it also leads to decreased ROS production in response to various stimuli [21]. Studies evaluating the relative roles of the peroxidase (GPx) and PLA_2_ activities of Prdx6 in pulmonary microvascular cells exposed directly to the oxidant (tert-butyl hydroperoxide) revealed that both activities participated in protecting cells from oxidative stress [22].

## 3. Peroxiredoxin 6 in Endothelial Mechanotransduction

The endothelium by virtue of its location “senses” the alteration of blood flow, as would occur with ischemia–reperfusion (I/R). Our work in the past decade has shown that the stop and restart of blood flow to the lung activates “flow-sensitive” machinery on lung endothelium comprising a K_ATP_ channel and NADPH oxidase 2 (NOX2) that leads to the production of ROS [23,24]. Our work has shown that the deletion of NOX2 (null mice) leads to a complete abrogation of ROS production with lung ischemia and reperfusion [25,26]. The deletion of Prdx6 (Prdx6-null mice) also led to diminution of ROS with lung ischemia [27]. This seemed paradoxical, as a lack of the antioxidant would be expected to increase ROS production. Our investigations revealed that Prdx6 was required for NOX2 activation. Indeed, we showed that in pulmonary microvascular endothelial cells, the phospholipase activity (PLA_2_) of Prdx6 was a key event in NOX2 activation [11,28]. 

However, Prdx6 does act as an antioxidant in other models of I/R that involve systemic organs [29], for instance, in liver I/R, where ROS production is believed to be predominantly via mitochondrial ROS, deletion of Prdx6, and increased injury. Isolation of mitochondria after ischemia or I/R demonstrated that a lack of mitochondrial Prdx6 led to mitochondrial dysfunction via disruption of mitochondrial respiration [29]. In a model of intestinal I/R (achieved by occlusion of the superior mesenteric artery), Prdx6 was found to play a protective role as delivery of exogenous Prdx6 significantly reduced I/R injury. However the administration of mutant forms of Prx6 (Prx6C47S) that do not possess the peroxidase activity had no protective effect [30]. Elsewhere, a hind limb model of I/R was found to show reduced injury upon exogenous administration of Prdx6 [31]. In a cerebral ischemia–reperfusion model, where non-endothelial cells were also evaluated, blocking Prdx6–PLA_2_ (either by siRNA or by a PLA2 inhibitor MJ33) reduced pro-inflammatory cytokines, and increased neuronal survival was compared to the wild type. In this study, both in vitro (oxygen–glucose deprivation/recovery) and in vivo (middle cerebral artery occlusion) models were used and I/R represented hypoxia–reoxygenation [32]. Thus, in several systemic models of I/R, Prdx6, either in endogenous form or administered exogenously, neutralizes oxidative stress, thus reducing the extent of tissue injury destruction with I/R. Overall, in the models of systemic I/R, the antioxidant effect of Prdx6 was significant, while lung I/R showed Prdx6–PLA_2_ activity. This discrepancy arises because systemic I/R models differ greatly from lung I/R—in the lung, the stop of blood flow does not compromise oxygen supply as the lung parenchyma obtains oxygen from the alveolus, but the systemic organs are dependent on blood flow for oxygen supply [33]. Lung I/R is thus a mechanotransduction event, where endothelial signaling with alteration of flow occurs due to loss of the mechanical component of flow and is independent of the partial oxygen pressures. In contrast, systemic I/R represents both altered flow stimulus and the effects of anoxia or hypoxia, followed by reoxygenation. The ROS generated by lung I/R has been reported to be via NADPH oxidase 2, whose assembly is dependent on the PLA_2_ activity of Prx6 [11,27]. Thus, loss of Prdx6 compromises ROS production with mechanotransduction. In systemic I/R, ROS is produced via either xanthine oxidase or the disrupted mitochondrial electron system (complex I–complex III) [34,35]. These pathways do not seem to involve Prdx6. Thus, in metabolically active organs, lack of Prdx6 leads to enhanced I/R injury, while in lungs where normoxia is maintained throughout I/R, and where the ROS production occurs via Prdx6–PLA_2_ activity, lack of Prdx6 leads to compromised ROS production [27].

## 4. Peroxiredoxin 6 in Endothelial Chemotransduction

Pulmonary endothelial cells are exposed to inhaled agonists via the alveolar capillary interface or agonists in the systemic circulation. Exposure to paraquat (PQ), a toxic herbicide, results in cell death of lung endothelial cells [36,37,38]. PQ, a prototypical redox cycling agent, is rapidly taken up and accumulates in endothelial cells. It produces ROS, leading to oxidative stress and apoptosis by a mitochondrial dependent pathway [39]. Pulmonary endothelial cells, when exposed to PQ, show a significant increase in oxidative damage and cell death [40]. In Prdx6-null mice, PQ administration led to increased lung injury. Although the effect on the pulmonary endothelium was not evaluated per se, vascular leakiness, an index of endothelial injury, showed a significant increase in Prdx6-null mice treated with PQ [36]. This indicates that Prdx6 is pivotal in protection, presumably via its peroxidase activity, against PQ. 

**Figure 1:**
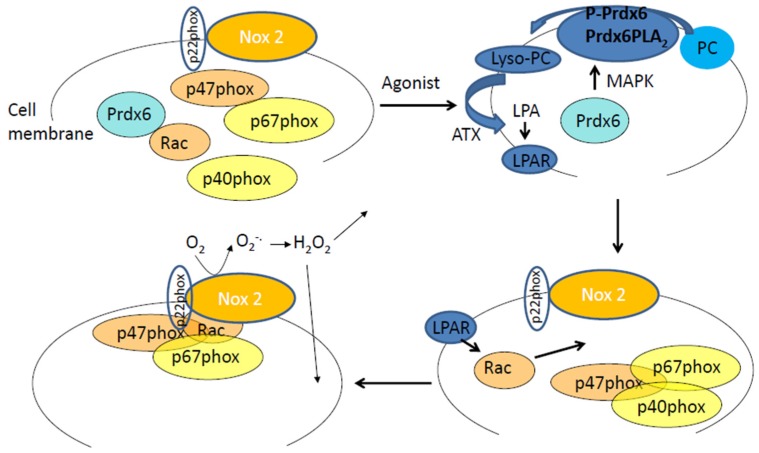
The Prdx6–PLA_2_ activity and the production of reactive oxygen species (ROS) by endothelial cells in response to an agonist. In the cytosol are Prdx6 and cytosolic subunits of NADPH 2 oxidase (p40phox, p67phox, p47phox, and Rac). Upon phosphorylation (in response to an agonist, Angiotensin II, or LPS) Prdx6 translocates to the plasma membrane. P-Prdx6 has phosphospholipase A_2_ activity via which it hydrolyses membrane phosphatidylcholine (PC) to lysophosphatidylcholine (lyso-PC). Lyso-PC is catalyzed to lysophosphatidic acid (LPA) by the enzyme autotaxin (ATX). LPA binds to its receptor on the cell membrane, and the resulting signaling cascade leads to Rac phosphorylation that in turn enables assembly of the cytosolic components of NADPH oxidase 2 with the membrane bound components (gp91phox or Nox2 and p22phox). The assembled enzyme reduces molecular oxygen to superoxide which then dismutates to hydrogen peroxide. H_2_O_2_ can participate in extracellular and intracellular signaling cascades.

Chemotransduction, associated with the endotoxin LPS, involves a role for Prdx6–PLA_2_ activity [41]. LPS is a component of the outer membrane in Gram-negative bacteria and is involved in the pathogenesis of sepsis [42,43]. We reported that LPS exposure on endothelial cells leads to ROS production via the NOX2 pathway [41]. This requires PLA_2_ activation of Prdx6. Indeed, we previously demonstrated that Prdx6–PLA_2_ generates lysophosphatidylcholine (LPC), which is converted to lysophosphatidic acid (LPA) by the lysophospholipase D activity of autotaxin. The binding of LPA to its receptor (LPAR) leads to an assembly of NOX2 components on the cell membrane [28]. Thus Prdx6–PLA_2_ activity facilitates (via LPA production) ROS generation by endothelial cells in response to agonists such as LPS (Figure 1). However, it is not clear if the PLA_2_ signaling pathway is effective in NOX2 activation only within the same cell. Studies elsewhere have shown that PLA_2_ products, such as eicosanoids and platelet-activating factor engage receptors in a paracrine fashion [44]. Other cells, such as PMN, also show Prdx6–PLA2 activity and, therefore, it is possible that PLA_2_ products secreted from non-endothelial and endothelial cells can activate NOX2 in a paracrine manner. Investigations into intercellular cross talk via PLA_2_ products have not been reported as yet. Our studies monitored PLA_2_ and NOX2 activation in entire endothelial monolayers; such methods are insufficient to detect the local and paracrine effects of PLA_2_ products, therefore, the NOX2 activation within each cell type of our ROS measurements, and thus, to an extent, the PLA_2_ cleavage. The methods we used showed a conclusive link between Prdx6–PLA_2_ activity; indeed we demonstrated that a lack of Prdx6–PLA_2_ activity, as achieved by mutations in Asp140, led to reduction in ROS [28].

In vivo exposures to LPS either via the systemic circulation or intratracheal instillation also resulted in ROS generation by the lung endothelium via the NOX2 pathway. Deleting Prdx6 by the use of Prdx6-null mice or by the inhibitor MJ33 resulted in a decrease in endothelial ROS production [41,45]. However, in other models of LPS-induced systemic injury, such as kidney damage, that involve an inflammatory response, Prdx6 seemed to be participating in a protective role. Prdx6-overexpressing mice showed decreased mortality and renal injury following the LPS challenge, compared to wild type (WT) mice. The inflammatory response of the kidney to LPS in the form of infiltration of macrophages, T-cells, and neutrophils, were also lower in Prdx6-overexpressing mice, as compared to wild-type mice [46]. This indicated that LPS-induced chemotransduction involved ROS generation (Prdx6–PLA_2_ activity) in certain models of injury, while in others, it was the antioxidant activity of Prdx6 (peroxidase activity) that was observed in response to ROS generation that presumably occurred via non NOX2 pathways.

Angiotensin II (Ang II) is an endogenous agonist that raises blood pressure primarily through vasoconstriction by acting on the endothelium and producing ROS [47]. We and others showed that Ang II activates the NOX2 pathway on endothelial cells in vitro and on the lung endothelium in vivo, via Prdx6–PLA_2_ activation [11,28]. Blocking PLA2 activation can potentially provide a pharmacological approach to Ang II-induced vasocontriction.

**Figure 2:**
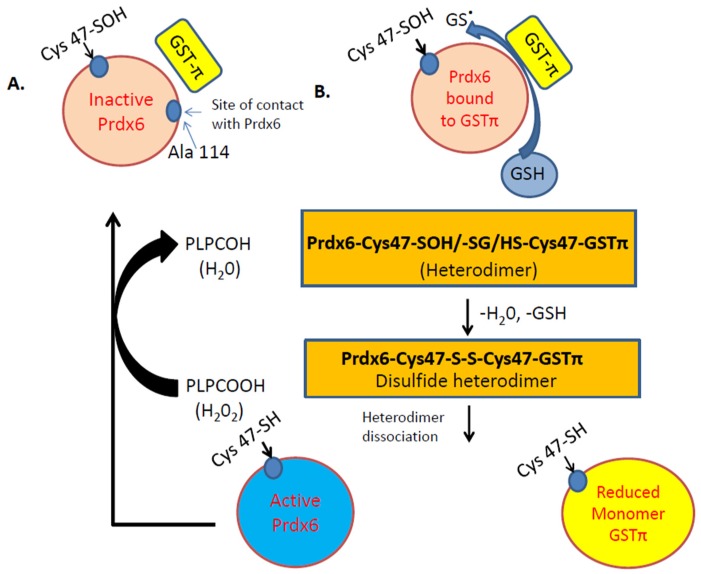
Illustration of the Peroxidase activity of Prdx6. A. Inactive Prdx6: its active site (Cys 47) needs to be oxidized to Cys-sulfenic acid (Cys47-SOH). B. After oxidation of Cys47 to sulfenic acid (-SOH), the Prdx6 forms a heterodimer with thiolate anion (via GSTπ). S-glutathionylation of the heterodimer, followed by its alignment with the catalytic Cys47 of GSTπ, results in the formation of a disulfide-based heterodimer. Reduction of this disulfide bond by GSH causes heterodimer dissociation to active Prdx6 monomer. The active monomer reduces phospholipid hydroperoxide (PLPCOOH) to PLPCOH.

A unique feature of Prdx6 is the regulation of its antioxidant action by partner protein glutathione S-transferase Pi (GSTπ). GSTπ is a member of the phase II detoxification enzyme family that catalyzes the formation of thioether bonds between GSH and electrophilic centers on small proteins. One such protein is Prdx6 and GST-π is well established to catalyze the conjugation of the antioxidant GSH to Prdx6 [7,48]. GSH is not able to access the catalytic cysteine residue on Prdx6. Upon conversion to its anionic form, GS- (by GSTπ), it can bind to Prdx6 (Figure 2). The glutathionylation of the oxidized cysteine in Prdx6 is followed by a spontaneous reduction of the mixed disulfide and restoration of enzymatic activity of Prdx6 (Figure 2B) [7]. GSTπ has been reported to be highly expressed in endothelial cells, specifically with pathologies such as neurological disorders [49], and protects against endothelial permeability and damage in response to inflammatory stimuli [50]. Studies using several oxidant and inflammation stimuli have demonstrated that GSTπ acts as a negative regulator of endothelial dysfunction [51]. These studies did not investigate the role of Prdx6 per se; however, based on the fact that GST-π can exert its protective function via activation of the oxidized Prdx6 [48], it is presumable that Prdx6 plays a role in protection against endothelial dysfunction in oxidant-induced pathologies.

## 5. Peroxiredoxin 6 in Inflammatory Response

ROS-induced signaling has been well established as playing a role in the onset and amplification of inflammation. The endothelium is a converging site of inflammation, where PMN, macrophages, and other immune cells adhere to and extravasate from blood to tissue. An enhanced ROS generation on the endothelium is thus a first step in inflammation [52]. We showed that ROS production by the endothelium in response to either chemotransduction or mechanotransduction is an inflammatory stimulus [53]. Endothelial ROS leads to an increase in cellular adhesion molecules and pro-inflammatory cytokines that recruit PMN and other immune cells to the vascular wall. ROS produced by PMN in addition to the endothelial ROS, leads to oxidative stress in the vasculature and leads to opening of inter-endothelial cell–cell junctions that in turn promotes the migration of PMN, etc., across the vascular wall [52]. Thus, enzymes with roles in redox balance, i.e., with both antioxidants as well as ROS-generating capacity, play a critical role in inflammatory and immune responses. Prdx6 possesses both of these activities and, therefore, it is reasonable to assume that this enzyme would regulate inflammation in vivo.

Upregulation of Prdx6 has been observed under conditions of increased ROS generation in various models of injury, implying that oxidative stress leads to increased Prdx6 transcription. This also implies that Prdx6 expression can be indicative of oxidative stress pathologies. Studies in patients with peripheral arterial disease show that circulating levels of PRDX1, 2, 4, and 6 are markedly raised [54]. In patients with abdominal aortic aneurysms, there are increased levels of Prdx6, both in the plasma and in the tissue from the aneurysm [20]. Lack of Prdx6 (PRDX6-null mice) showed significantly higher aortic lesions in response to oxidative stress [55]. Prdx6 is also high in serum of patients with osteoarthritis and those with femoral neck fracture and there are strong correlations between the levels of these molecules in the serum and severity of these conditions [56]. It is not clear in all of these models of inflammation whether Prdx6 participates in redox imbalance through protective antioxidant functions or by redox signaling via activation of NOX2. In an oxidative stress environment, hyperoxidation of Prdx6 is reported to upregulate its PLA2 activity [57]. Conversely, environments with a high inflammation and oxidative stress load, such as aortic lesions or aneurysms, can cause post-translation modification of Prdx6, such that it loses its antioxidant activity [58]. The major pro-inflammatory transcription factor NFκB has been observed to be regulated by Prdx6 [19]. The Prdx6 promoter (−1139 bp) containing κB binding sites, showed reduced promoter activity in Prdx6 null cells [59]. Besides the peroxidase activity that facilitates protection from oxidative stress, Prdx6 also protects by downregulating NFκB [60].

## 6. Prdx6 in Wound Repair

Wound repair is based on the balance between oxidative stress and regeneration. Thus, most of the injury- and repair-regulated genes encode for antioxidant enzymes, which scavenge on ROS [61,62]. Among the wound-regulated genes that have been identified, one is Prdx6 [61]. In mice, skin wounds were found to have an overexpression of Prdx6 in the epidermis [63]. The epidermis of psoriatic patients and cells of the wound granulation tissue also showed high expression of Prdx6 [61,63].

Prdx6 seems to be protective against dermal injury and to facilitate wound repair. Indeed, mice overexpressing Prdx6 in the epidermis were protected from UVA- and UVB-induced skin damage [61,63]. These mice also showed accelerated wound closure [64]. Similarly, lack of Prdx6, i.e., Prdx6-null mice, increased keratinocyte apoptosis and endothelial damage. In another study using an excision model of injury, the role of the Prdx6 in the endothelium was found to be crucial in the repair of endothelial cells damaged by oxidative stress [21]. Prdx6-null mice showed the appearance of granulated tissue associated with hemorrhage due to endothelial damage. Using chimeric mice (wild-type and Prdx6-null mice with WT- and Prdx6-null bone marrow cells) and ultrastructural analysis of tissue, it was observed that Prdx6 expression on the endothelium correlated with the formation of new blood vessels, suggesting that Prdx6 is required for endothelial cell integrity and viability in the wound tissue [21]. The onset of hemorrhage correlating with the formation of novel blood vessels suggests the importance of Prdx6 for endothelial cell maintenance, structure, and viability in wounded tissue. However, lack of Prdx6 affected the endothelial cell integrity, structure, and function, only during oxidative stress and not under normal conditions, implying that the protective role was crucial only post-injury and did not affect the endothelium under basal conditions. Overall, these studies seem to reveal the role for the peroxidase activity of Prdx6 in protecting the epidermis from oxidative damage. However, it is not clear if peroxidase and/or PLA_2_ activities might participate in wound repair. This is because transcription factors, such as NFκB, that are crucial for skin wound repair processes are regulated both by ROS and Prdx6 [65] and there is some evidence for the role of Prdx6–PLA_2_ activity in NFκB regulation [59].

## 7. Prdx6 in the Pathogenesis of Diabetes

Increased oxidative stress appears to be a major factor leading to insulin resistance and pathogenesis of diabetes [66]. Pancreatic β-cells that produce insulin express very low levels of antioxidant enzymes and are thus susceptible to oxidative stress [67]. Although β-cells express Prdx6 [68], studies using insulin-producing RINm5F cells have shown that Prdx6 expression (both mRNA and protein) is downregulated in response to proinflammatory cytokines [69]. This reduces the antioxidant capacity of Prdx6. Furthermore, lack of Prdx6 (in mice) causes the development of a phenotype similar to early-stage Type II diabetes in terms of both reduced glucose-dependent insulin secretion and increased insulin resistance [70].

Dysfunction of the endothelium is similarly observed in diabetes [71]. Under conditions of oxidative stress, the endothelial repair mechanisms are hindered. This is because endothelial repair occurs via endothelial progenitor cells (EPC) [72]. Under basal conditions, EPCs are immature cells and differentiate into mature endothelial cells. With vascular injury, EPC mobilization from the peripheral circulation (aided via growth factors and cytokines) to sites of damage is a crucial component of repair and angiogenesis. Both type 1 and type 2 diabetics have less circulating EPCs than matched healthy subjects. Diabetic EPCs also show reduced proliferation, adhesion, migration, and impaired development of tubules for the formation of a vascular network [71,73].

As oxidative stress and insulin resistance are associated with a proinflammatory state, inflammation often accompanies diabetes [71]. The link between diabetes, inflammation, and Prdx6 is not clear. On one hand, inflammation can often lower expression of Prdx6, while on the other, it is overexpressed in some inflammatory pathologies, such as inflammatory bowel disease (IBD) [74]. Indeed, human colonic biopsies of IBD patients showed high expression of Prdx6. Mice that lack Prdx6 show a marked increase in proinflammatory cytokines (IL-1β and TNF-α) and in matrix metalloproteases (MMP-2 and -9) in response to an inflammatory stimulus [75]. Elsewhere, these mice showed an insulin resistance phenotype [70]. Insulin resistance-associated inflammation could be partly regulated by the Prdx6, although the role of its PLA_2_ versus peroxidase activities in this regulation has not been clearly defined.

A recent report seems to point to a role for Prdx6 in glucose homeostasis, primarily via its antioxidant role [69]. Reports elsewhere indicate that the pathophysiological impairments associated with diabetes, such as sarcopenia (loss of muscle mass), muscle atrophy, and diabetic myopathy can potentially be rescued by Prdx6 [76]. Gene expression of MyoD and myogenin, that participate in the differentiation of muscle cells, was observed to be significantly lower in Prdx6-null mice as compared to wild type controls [76]. This points to a role for Prdx6 in the maintenance of muscle mass and points to a possible use of this enzyme for therapy in diabetic myopathy and sarcopenia. Thus, Prdx6 could potentially represent diabetes susceptibility in humans. Moreover, associations between Prdx6 expression and inflammatory endothelial phenotype are possible factors in endothelial dysfunction with diabetes.

## 8. Conclusions

**Figure 3:**
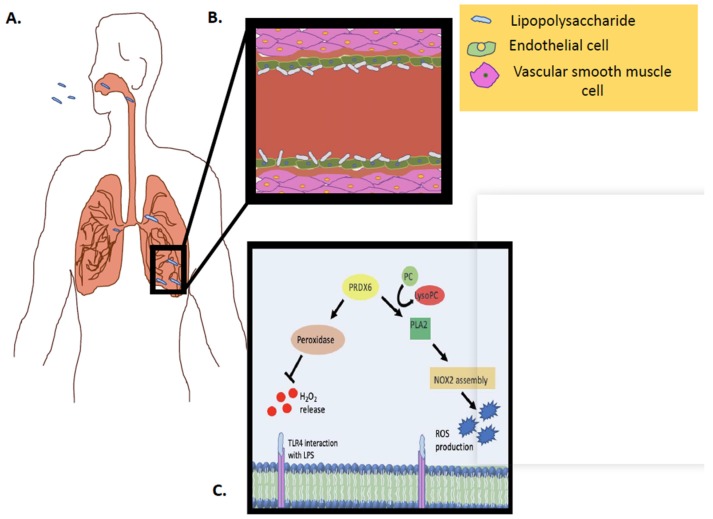
Schematic representation of Prdx6 activity (PLA_2_ and peroxidase) in vivo, in response to an inflammatory stimulus. **(A**) LPS inhalation and interaction with pulmonary capillary. (**B**) LPS inhalation and interaction with endothelial cells in pulmonary capillary. (**C**) LPS interaction with the endothelial cell membrane and the Prdx6–PLA2 activity as well as the Prdx6–peroxidase activity.

The family of peroxiredoxins has been recognized as major scavenging enzymes in mammalian systems. Of these, Prdx6 is unique in that its peroxidase activity facilities oxidant scavenging, while Prdx6–PLA2 activity is crucial in oxidant generation (Figure 3). The dual function of this enzyme presumably enables it to act as a “regulatory body”, whereby its protective role and its oxidant-generating action is not exceeded, so as to perturb vascular homeostasis. This optimal regulation of Prdx6 expression and activity is crucial in fine tuning cellular ROS levels and seems to occur via complex signaling cascades that involve NFκB and oxidants. While upregulation of Prdx6 is protective, it is also associated with several pathologies ranging from aortic lesions to diabetes susceptibility. Low levels or lack of Prdx6 are also associated with inflammatory diseases. These observations and reports indicate that regulation of the peroxidase and PLA_2_ activities is complex and intersects with several other transcriptional pathways. Further studies using Prdx6 mutants and null systems on diverse models of inflammation and injury are needed to strengthen our knowledge regarding the relationship between Prdx6 regulation and oxidant and antioxidant signaling and the final physiological and pathophysiological effects.
